# An All-Suture–Based Technique for Meniscal Repair Is Cost-Effective in Comparison to Partial Meniscectomy for Horizontal Cleavage Tears

**DOI:** 10.1016/j.asmr.2023.100847

**Published:** 2024-02-13

**Authors:** Seth L. Sherman, Neil Askew, Leo M. Nherera, Richard J. Searle, David C. Flanigan

**Affiliations:** a Department of Orthopaedic Surgery, Stanford University School of Medicine, Redwood City, California, U.S.A.; b Smith & Nephew, Fort Worth, Texas, U.S.A.; c Sports Medicine and the Department of Orthopaedics, The Ohio State University Wexner Medical Center, Columbus, Ohio, U.S.A.

## Abstract

**Purpose:**

To determine the cost-effectiveness of meniscal repair (MR) using an all-suture–based technique when compared to partial meniscectomy (PM) for horizontal cleavage tears (HCTs) from a payor’s perspective in the United States.

**Methods:**

A state-transition model and cost-utility analysis were developed from a US payor’s perspective to project treatment costs and quality-adjusted life-years (QALYs) in a cohort of 35-year-old patients without osteoarthritis at baseline and presenting with either a lateral or medial HCT. Two outpatient costing perspectives were used, namely ambulatory surgical centers (ASCs) and hospitals. The state-transition model had 7 health states with transition probabilities, costs, and utilities obtained from the existing literature. Cost-effectiveness was assessed using a willingness-to-pay threshold of $100,000/QALY, and sensitivity analysis considered the effects of parameter uncertainty on model results. MR failure rates were focused on an all-suture–based technique; however, in a separate scenario, this study considered effectiveness data from various MR techniques and devices.

**Results:**

MR dominated PM over a lifetime horizon, increasing QALYs by 0.43 per patient and decreasing the cost by $12,227 per patient within a hospital setting (and by $12,570 within an ASC). MR with an all-suture–based technique continued to be the dominant treatment when age at primary treatment was varied between 30 and 60 years. Sensitivity analysis showed that MR was not cost-effective in year 1, was cost-effective from year 2, and was cost-saving from year 6 onward from both ASC and hospital perspectives. Probabilistic sensitivity analysis found that MR was cost-effective over a lifetime horizon in 99% of 10,000 iterations on base-case analysis.

**Conclusions:**

Using a lifetime horizon, this study found that from a payor’s perspective, MR is a cost-saving intervention when compared with PM in patients with an HCT.

**Level of Evidence:**

Level III, economic analysis.

Horizontal cleavage tears (HCTs) of the meniscus are situated parallel to the tibial plateau and divide the meniscus into inferior and superior portions.^1^ HCTs are commonly encountered orthopaedic injuries, and by some estimates HCTs comprise up to one-third of meniscal tears.^2^ Compared with other common types of meniscal tears, HCTs have an increased incidence and severity of chondral lesions.^3^

Unlike vertical-longitudinal tears, which have been deemed ideal candidates for meniscal repair (MR), HCTs have traditionally been treated either conservatively or with partial or total resection.^4^, ^5^, ^6^, ^7^, ^8^, ^9^, ^10^, ^11^ This treatment pathway has largely been determined based on concerns surrounding the technical difficulty of HCT repair, healing rates, and potential suture failure due to mechanical stresses.^12^

However, data from the past decade have shifted clinical consensus after it was shown in the literature that MR can yield encouraging results and reduce the likelihood of osteoarthritis (OA) developing in patients, which in turn may reduce the chance that a total knee replacement (TKR) will be required in the longer term. A 2014 systematic literature review of 9 studies (totaling 98 HCTs) reported a combined success rate of 78% after an MR,^12^ whereas meniscectomy typically results in a lower revision rate in the short term. However, it is important to consider that patients receiving meniscectomy will have a higher risk of OA that also has higher resource implications, including future surgery (e.g., TKR).^13^

A systematic review (including 16 studies) found that MR is the most cost-effective intervention for reparable meniscal tears.^14^ There have been several long-term cost-utility analyses in this area.^15^, ^16^, ^17^, ^18^ All the cited studies have shown that the benefits of reducing OA cases and future TKRs when choosing MR over partial meniscectomy (PM) outweigh the cost of a higher revision rate for other types of tears, and thus, this study seeks to explore whether this finding holds for HCTs.

The purpose of this study was to explore the costs and associated outcomes of MR and PM for HCTs to determine their relative cost-effectiveness. We hypothesized that MR would be cost-effective in comparison to PM in the treatment of HCTs.

## Methods

### Model Structure

**Fig 1:**
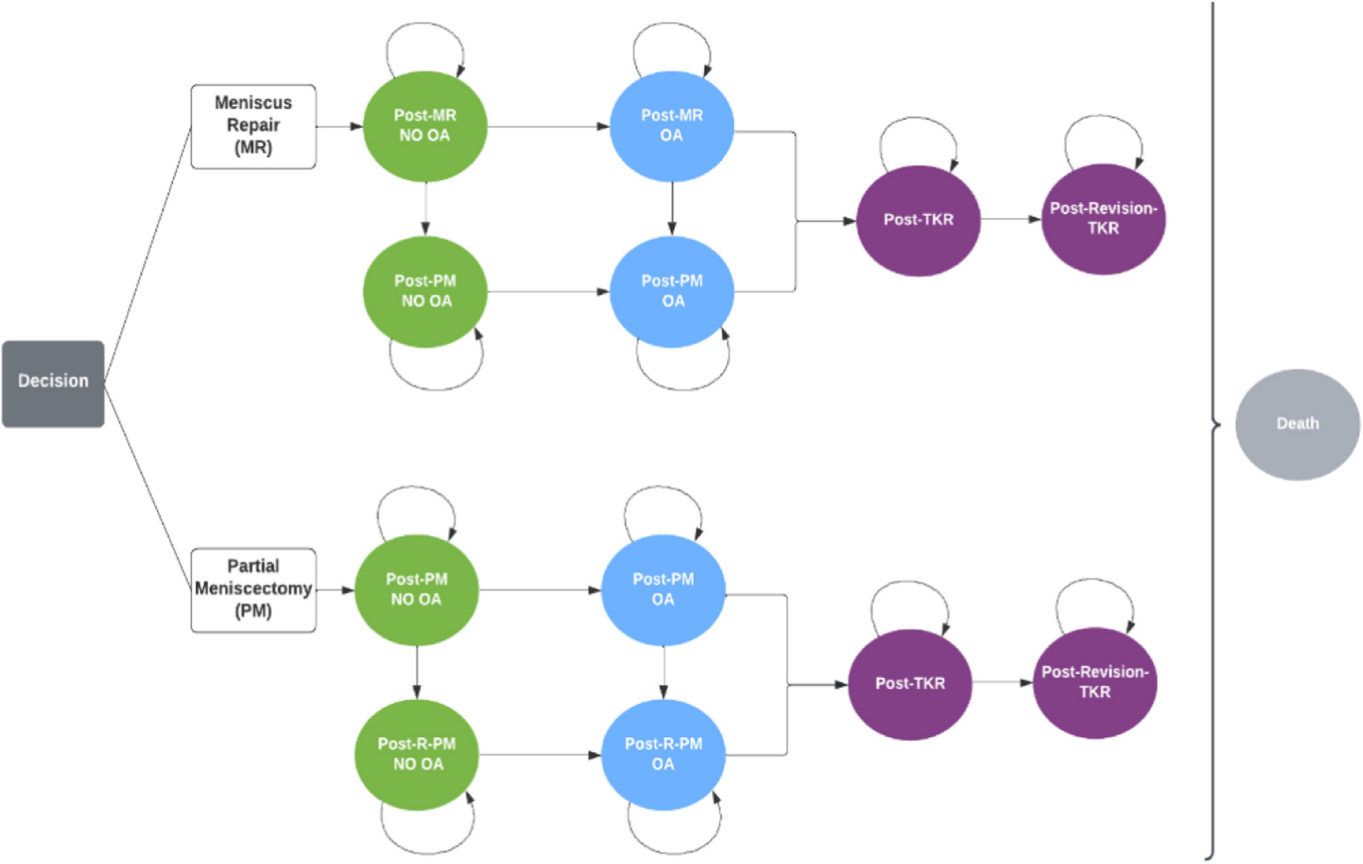
Markov model structure for cost-utility analysis of meniscal repair (MR) versus partial meniscectomy (PM). (OA, osteoarthritis; TKR, total knee replacement.)

As with any economic evaluation, a key step is to select a suitable comparator to benchmark the new intervention being considered.^19^ This analysis used a prospective, multicenter trial^19^ that centered on HCTs to consider whether MR is cost-effective when compared with PM. The model structure was informed by several other economic evaluations that have compared MR with PM or total meniscectomy.^15^, ^16^, ^17^, ^18^ Faucett et al.^15^ used a Markov model, and this was considered the most appropriate model structure because it allows for procedure revision—or the development of OA—to occur independently and at multiple time points. As shown in Figure 1, in both the intervention (MR) and comparator (PM) arms of the model, patients start after their primary treatment. After a primary procedure, there is the possibility of revision, which is a second PM procedure if this was the patient’s primary treatment or a patient’s first PM if he or she received an MR as his or her primary treatment.

By use of a similar model structure to that in the previous economic evaluations, a cost-utility analysis was performed and is a preferred type of health economic evaluation in medicine that compares projected costs and health outcomes of alternative treatments. As a measure of health outcome, effectiveness was measured in quality-adjusted life-years (QALYs) ranging from 0 (death) to 1 (perfect health). In a Markov model, outcomes can occur over time and patients move between mutually exclusive health states. In addition, a cohort of simulated participants is initially allocated to each treatment strategy and subsequently assigned to health states based on the estimated transition probabilities over a defined cycle length.

In the base case, patients started in the “no OA” health state having undergone an MR or PM, and each patient had an intervention-specific probability to progress to symptomatic knee OA. As shown in Table 1, time-specific OA progression rates for PM came from Rockborn and Messner^21^ for up to 10 years and from Hulet et al.^22^ for over 10 years after treatment. A relative risk (RR) of 0.55 (MR vs PM) was obtained from a meta-analysis of 5 studies that we conducted comparing MR with meniscectomy for standard meniscal tears^23^, ^24^, ^25^, ^26^, ^27^ and was used as the base case to adjust patients’ possible transition to OA. Although our preference would have been an RR specific to HCTs as opposed to meniscal tears in general, data limitations meant that this was not possible for this study. However, several studies have explored this issue and found similar OA progression irrespective of tear type.^28^^,^^29^

**Table 1:** Demographic Characteristics and Transition Probabilities

	Base Case	PSA		Source
		Range	Distribution	
Patient demographic characteristics				
Primary treatment age, yr	35	Not varied in PSA	NA	Assumption
Female, %	50	Not varied in PSA	NA	Assumption
Proportion with OA at baseline, %	0	Not varied in PSA	NA	Assumption
Mortality				
Baseline mortality for ages 35-110 yr, % probability	0.21-52.29	NA	NA	Social Security Administration
PM, MR, TKR, and R-TKR mortality risk, % probability	0.3	±20%	Beta	Faucett et al.^15^ (2018) and Feeley et al.^16^ (2016)
Index procedure revision or failure rates				
Annual failure rate of PM and transition to TM, % probability	0.2	±20%	Beta	Calculated from 2-yr rate presented by Shanmugaraj et al.^33^ (2019)
Annual failure rate of MR and transition to TM, % probability				
MR with all-suture technique	9.12	±20%	Beta	Calculated from 2-yr rate presented by Kurzweil et al.^20^ (2021)
MR with various techniques	2.84	±20%	Beta	Calculated from 2-yr rate presented by Shanmugaraj et al.
Transition to OA				
Annual probability of OA, %				
Year 1-5 after PM	11.27	±20%	Beta	Converted from 5-yr endpoint in Rockborn and Messner^21^ (2000)
Year 6-10 after PM	3.51	±20%	Beta	Converted from 10-yr endpoint in Rockborn and Messner
Year ≥11 after PM	0.89	±20%	Beta	Converted from 15-yr endpoint in Hulet et al.^22^ (2015)
RR of Transition to OA				
RR of OA development (MR compared with PM)	0.55	±20%	Log-normal	Internal meta-analysis
Conversion to TKR from OA				
Annual probability of conversion from symptomatic OA to TKR, %	2.27	±20%	Beta	Rogers et al.^17^ (2019) based on 2019 data from United Healthcare (Minnetonka, MN)
Conversion to R-TKR from TKR				
Annual R-TKR or TKR Failure %				
Year 1-4	1.9	±20%	Beta	Feeley et al. and Bedair et al.^30^ (2014)
Year 5-9	1	±20%	Beta	Feeley et al. and Bedair et al.
Year 10	0.9	±20%	Beta	Feeley et al. and Bedair et al.
Year ≥11	0.6	±20%	Beta	Feeley et al. and Bedair et al.

MR, meniscal repair; NA, not applicable; OA, osteoarthritis; PM, partial meniscectomy; PSA, probabilistic sensitivity analysis; RR, relative risk; R-TKR, revision total knee replacement; TKR, total knee replacement; TM, total meniscectomy.

Conversion from OA to TKR occurred at an annual rate of 2.27% for patients in both arms of the model used by Rogers et al.^17^ Table 1 shows the inputted values for 1 possible revision TKR that vary between 1.9% and 0.6% annually depending on the time since primary treatment.^16^^,^^30^ The risk of failure or revision was only applied for the first 3 cycles or years of the model projections because beyond this point, it is difficult to associate the need for reoperation with the primary treatment.^31^ Table 1 details the transition probabilities and patient demographic characteristics used in the model.

After a primary procedure and possible revisions, the model projected strategy-specific progression to symptomatic OA, TKR, and revision TKR in a cohort of 35-year-old patients presenting with HCTs and no OA at the time of treatment. The model starting age of 35 years was based on the average age of patients in the study of Kurzweil et al.,^20^ from which we sourced the failure rates for MR. An assumption was made that patients could only transition to TKR from the OA health state; however, the number of TKR revisions was limited to 1 as shown in Figure 1. The arrows that point toward “Death” indicate the time-specific probability of mortality occurring from any subsequent health state (as reported in Table 1).

This model used annual cycles (Table 2), where individuals were reassigned between health states each year and the respective costs and/or utilities were discounted by 3% to adjust outcomes depending on the time they take to occur.^19^ A further time adjustment was also required to account for the discrete-time nature of this model. For example, without adjustment, costs and QALYs would be calculated based on outcomes at the start or end of an annual cycle. As has been discussed in the literature, an unbiased estimate of outcomes would assume that events occur halfway through a cycle (i.e., midway through a year in this analysis).^19^ Adjusting a discrete-time Markov model such that utilities and costs occur in the middle of a cycle is known as half-cycle correction and involves adding a cycle of half duration at the beginning of the process. Current health economic guidelines recommend using this correction,^32^ and hence, this is the approach used in our analysis. For validation purposes, the trapezoidal rule, which is another commonly used method for within-cycle correction, was also used and generated the same results as half-cycle correction.

**Table 2:** Costs, utility values, and disutility values

	Base Case	PSA		Source
		Range	Distribution	
Costs (per procedure)				
Index MR, 2021 $				
Hospital	4,996	±20%	Gamma	Medicare 2021 reimbursement costs plus NovoStitch Pro ASP
ASC	3,494	±20%	Gamma	Medicare 2021 reimbursement costs plus NovoStitch Pro ASP
Index and revision PM or TM, 2021 $				
Hospital	3,433	±20%	Gamma	Medicare 2021 reimbursement costs
ASC	1,931	±20%	Gamma	Medicare 2021 reimbursement costs
TKR, 2021 $	38,916	±20%	Gamma	Feeley et al.^16^ (2016) adjusted from 2014 prices
Revision TKR, 2021 $	46,698	±20%	Gamma	Feeley et al. adjusted from 2014 prices
Costs (per cycle)				
Nonoperative treatment of OA per year (also applied to post-TKR and post–revision TKR health states), 2021 $	3,486	±20%	Gamma	Feeley et al. adjusted from 2014 prices
Utilities (per cycle)				
No OA health state	0.9	0.72-1	Beta	Faucett et al.^15^ (2018) and Feeley et al.
OA health state	0.69	0.55-0.83	Beta	Faucett et al. and Feeley et al.
Post-TKR	0.835	0.68-1	Beta	Faucett et al. and Feeley et al.
Post–revision TKR	0.785	0.63-0.94	Beta	Faucett et al. and Feeley et al.
Disutility (per procedure)				
ME and MR	0.0077	±20%	Log-normal	Faucett et al. and Feeley et al.
TKR	0.0250	±20%	Log-normal	Faucett et al. and Feeley et al.
Revision TKR	0.0500	±20%	Log-normal	Faucett et al. and Feeley et al.
Other model parameters				
Cycle length	Annual	Not varied in PSA	NA	Assumption
Discount rate for costs, %	3	Not varied in PSA	NA	Assumption
Discount rate for QALYs, %	3	Not varied in PSA	NA	Assumption
Within-cycle correction	Half-cycle correction	Not varied in PSA	NA	Assumption

ASC, ambulatory surgical center; ASP, average sales price; ME, meniscectomy; MR, meniscal repair; NA, not applicable; OA, osteoarthritis; PM, partial meniscectomy; PSA, probabilistic sensitivity analysis; QALY, quality-adjusted life-year; TKR, total knee replacement; TM, total meniscectomy.

### Interventions and Cost Data

The MR procedure considered for the base-case analysis of this study was all-suture circumferential stitches using a self-retrieving all-inside suture-passing device (NovoStitch PRO Meniscal Repair System; Smith & Nephew, Andover, MA), with or without inside-out or outside-in hybrid repairs. This procedure is referred to as “MR all-suture” henceforth and forms our base case and main set of results. The use of this procedure and device was considered in a recent trial by Kurzweil et al.^20^ that reported a 2-year annual failure rate of 17.4% (4 of 23 patients); this was converted to an annual probability of transition to secondary PM of 9.12% (transition inputs are shown in Table 1). In a separate scenario analysis, we used MR failure rates from a systematic review and meta-analysis of 23 studies by Shanmugaraj et al.^33^ comparing treatment options for HCTs. For MR, this review combined various repair techniques (all-inside repair, open repair, inside-out repair, fibrin clot suture repair, refixation, and use of bioabsorbable arrows). Although the focus of this study was to compare MR all-suture with PM, this scenario was chosen to use data from a larger sample size and increase the generalizability of the results. Shanmugaraj et al. found that at 2 years of follow-up, 5.6% of primary MR procedures (13 of 232) failed to heal and required secondary meniscectomies. By use of these combined data, the annual probability of healing failure—in other words, the probability of transition to secondary PM—was 2.84%.

PM effectiveness data were also sourced from Shanmugaraj et al.,^33^ who reported a 2-year healing failure rate of 0.4% (2 of 476 patients), which as shown in Table 1 provided an annual probability of the need for a second PM of 0.2%. For both the MR and PM arms of the model, the chance of procedure revision or secondary PM was applied only in the first 3 years or cycles after primary treatment. Ronnblad et al.^31^ made the assertion that such failures after 3 years are more likely to be dependent on other factors such as new trauma or biological deficiencies rather than an unhealed repair. We relaxed this assumption in sensitivity analysis so that failure can occur at any point after a primary procedure to see the impact on our results.

Costs were estimated from the perspective of a US third-party payor, with Medicare reimbursement as a proxy for cost. When costs were obtained from the literature (Table 1), they were uplifted for inflation to present costs in 2021 prices.^16^ Utility values for no OA, OA, post-TKR, and post–revision TKR, as well as disutility values per procedure, are the same as those used by Faucett et al.^15^ and Feeley et al.^16^ and are displayed alongside the costs used in the model in Table 2. Outpatient procedure costs within hospital and ambulatory surgical center (ASC) settings were sourced directly from the Centers for Medicare & Medicaid Services 2021 procedure price lookup service.^34^

## Results

### Base-Case Results

This study projected that MR all-suture was associated with a higher revision rate when compared with PM in early model cycles; however, it was then followed by a reduced number of OA cases, TKRs, and revision TKRs in later model cycles. Over a lifetime horizon, the model estimated discounted cost savings of $11,838 ($41,845 vs $53,682) per patient for MR all-suture when compared with PM from a hospital perspective. The savings rose slightly to $12,182 per patient when using ASC reimbursement as a proxy for treatment cost. Discounted QALYs per patient increased by 0.43 QALYs (15.35 QALYs vs 14.92 QALYs), making MR all-suture dominant (i.e., the preferred treatment strategy) over a lifetime horizon. Table 3 displays the results for a cohort of 1,000 patients and shows that, using the base case, MR all-suture dominated PM over a lifetime horizon.

**Table 3:** Base-Case Results From Lifetime Horizon (Starting Age 35 Years) per 1,000 Patients

Outcome	PM	MR All-Suture	Difference
Total No. of revisions	6	248	+242
Total No. of OA cases	660	495	–165
Total No. of TKRs	366	268	–98
Total No. of revision TKRs	51	36	–15
Total QALYs	14,917	15,349	+431
Total costs, $			
Hospital outpatients	55,041,433	42,814,123	–12,227,310
ASC patients	53,529,984	40,960,005	–12,569,980
Base-case decision rule	MR all-suture dominates PM (additional QALYs and lower costs over lifetime horizon)		

ASC, ambulatory surgical center; MR, meniscal repair; OA, osteoarthritis; PM, partial meniscectomy; QALY, quality-adjusted life-year; TKR, total knee replacement.

### Sensitivity Analysis

Additional sensitivity analysis included varying parameters specified in Tables 1 and 2 (±20% from base value for most parameters) did not materially impact cost-effectiveness results. Longer follow-up horizons were consistently associated with greater cost reductions and QALY improvements for MR all-suture when compared with PM. The model found MR all-suture was not cost-effective in year 1, was cost-effective from year 2, and was the dominant strategy (increased QALYs at a lower cost) from year 6 from both ASC and hospital cost perspectives. In addition, a reduction in adverse events in terms of OA cases and resulting TKRs was seen when comparing PM with MR all-suture, as shown in Table 3. Results showing the starting age when other baseline attributes remained the same in the model are presented in Table 4; the ages selected were 30, 40, 50, and 60 years. Although increasing the age at primary treatment narrowed the difference in accumulated QALYs and costs, which is an expected result, Table 4 shows that MR all-suture remained a dominant strategy over a lifetime horizon.

**Table 4:** Results From Lifetime Horizon per 1,000 Patients (Varying Primary Treatment Age)

	PM	MR All-Suture	Difference
Primary treatment age 30 yr			
Total QALYs	15,731	16,184	+452
Total costs, $			
Hospital outpatients	58,097,259	45,103,161	–12,994,098
ASC patients	56,585,808	43,248,926	–13,336,881
Base-case decision rule	MR all-suture dominates PM (additional QALYs and lower costs over lifetime horizon)		
Primary treatment age 40 yr			
Total QALYs	14,010	14,418	+407
Total costs, $			
Hospital outpatients	51,636,428	40,277,706	–11,358,723
ASC patients	50,124,983	38,423,723	–11,701,260
Base-case decision rule	MR all-suture dominates PM (additional QALYs and lower costs over lifetime horizon)		
Primary treatment age 50 yr			
Total QALYs	11,955	12,306	+351
Total costs, $			
Hospital outpatients	43,760,434	34,465,323	–9,295,111
ASC patients	42,249,005	32,612,055	–9,636,951
Base-case decision rule	MR all-suture dominates PM (additional QALYs and lower costs over lifetime horizon)		
Primary treatment age 60 yr			
Total QALYs	9,651	9,934	+283
Total costs, $			
Hospital outpatients	34,899,475	27,995,863	–6,903,612
ASC patients	33,388,091	26,144,492	–7,243,599
Base-case decision rule	MR all-suture dominates PM (additional QALYs and lower costs over lifetime horizon)		

ASC, ambulatory surgical center; MR, meniscal repair; PM, partial meniscectomy; QALY, quality-adjusted life-year.

The RR reduction of OA development was used to calculate the OA risk of MR all-suture, which was equal to 0.55 in the base case. By use of a lifetime horizon, this resulted in MR all-suture remaining the dominant strategy until a value of 0.92, where it changed to cost-effective; then at 0.97, it was found to no longer be cost-effective when compared with PM at a willingness-to-pay (WTP) threshold of $100,000/QALY.

**Fig 2:**
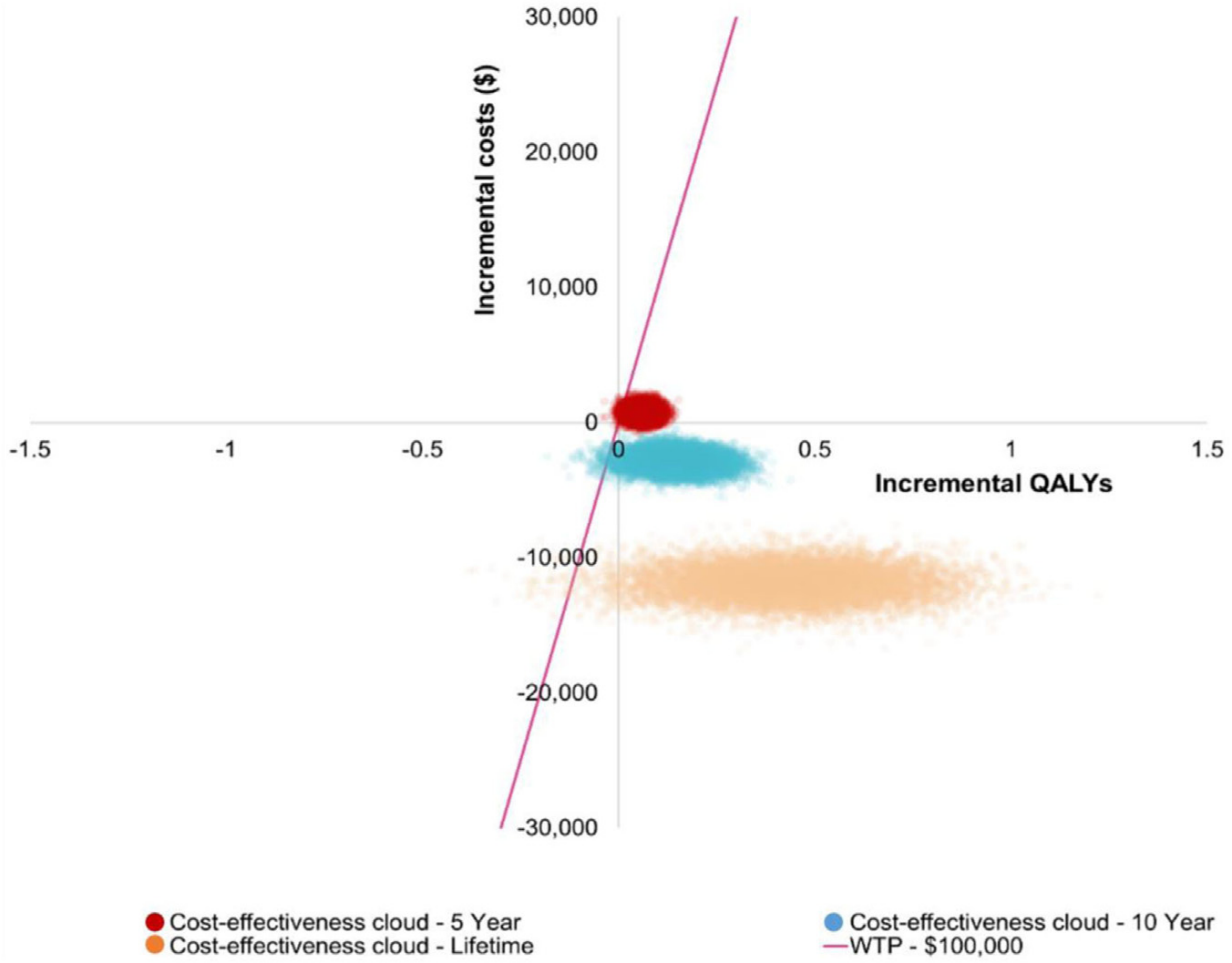
Cost-effectiveness plane (using hospital outpatient costs). (QALY, quality-adjusted life-year; WTP, willingness-to-pay threshold.)

A probabilistic sensitivity analysis (PSA), in which each parameter in a model is varied within its respective distributions simultaneously, was also conducted. For each iteration, differences in costs and QALYs were computed for the 2 interventions, and these are displayed as the cloud of orange dots in Figure 2, with each dot referring to an incremental cost-effectiveness ratio (ICER) from the US hospital perspective. Most of these dots or simulations fall within the bottom right-hand corner (reduced costs and increased QALYs) of the cost-effectiveness plane and thus deem MR all-suture as cost-saving with QALY gains over a lifetime horizon. Additionally, points that fall to the right of the red line consider MR to be cost-effective at a WTP threshold of $100,000/QALY. To summarize, the PSA found that 99.84% of iterations (orange dots in Fig 2) were cost-effective at this WTP threshold for a lifetime model horizon and from a US hospital perspective.

Figure 2 also displays the simulated ICER values for 5- and 10-year time horizons, which are shown as red and blue dots, respectively. This allows us to visually observe how most of these points show that MR all-suture remained cost-effective at a WTP threshold of $100,000/QALY when compared with PM for most simulations. In Figure 2, we can also observe that a small number of the simulated ICER points fall in the bottom left-hand quadrant, where PM accumulates more health benefit but also costs more. The WTP threshold dictates how much a payor might be willing to pay to obtain this additional health benefit and/or QALYs.

As mentioned in the “Methods” section, in addition to the base-case results comparing MR all-suture with PM, another scenario was run to replace MR all-suture data from Kurzweil et al.^20^ with effectiveness data from Shanmugaraj et al.^33^ that considered various MR techniques and devices. As set out in Table 1, this lowered the annual probability of failure for MR from 9.12% to 2.84% and, as a result, improved the favorability of MR when compared with PM for HCTs, increasing discounted cost savings to $15,063 per patient and discounted QALYs gained per patient to 0.51 from a US hospital perspective. Re-running the PSA increased the number of simulations where MR was cost-effective at $100,000/QALY when compared with PM from 99.84% to 99.89%.

As previously noted, the decision was taken to limit the time frame in which a revision could occur and be attributed to the primary treatment to 3 years.^31^ As part of the sensitivity analysis, this assumption was removed and did increase the number of MR all-suture revisions relative to PM. However, it did not significantly impact conclusions around cost-effectiveness. For instance, re-running the PSA after removing this assumption reduced the likelihood of MR being cost-effective when compared with PM from 99.84% to 99.37% at our selected WTP threshold of $100,000/QALY.

**Fig 3:**
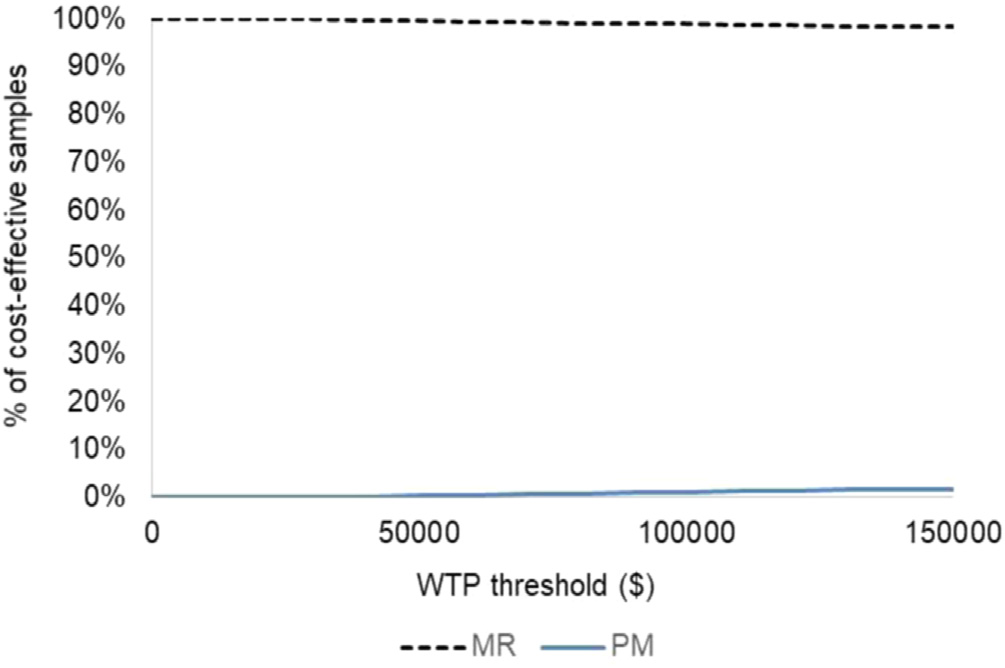
Cost-effectiveness acceptability curve: meniscal repair (MR) versus partial meniscectomy (PM). (WTP, willingness to pay.)

The sensitivity analysis findings taken together provide reassurance that the study results are robust because changing assumptions simultaneously does not impact the conclusions of the preferred strategy in terms of cost-effectiveness. The WTP threshold was also varied between $0 and $150,000, and the result is shown as a cost-effectiveness acceptability curve in Figure 3. This finding shows that there is a low likelihood that selecting MR would be the wrong choice from an economic standpoint.

## Discussion

In this study, we found that from a payor’s perspective, MR is a cost-saving intervention when compared with PM in patients with HCTs over a lifetime. Our sensitivity analysis results showed that MR was not cost-effective in year 1, was cost-effective from year 2, and was cost-saving from year 6 onward from both ASC and hospital cost perspectives. The favorable results for MR can be explained by the reduction in OA cases and therefore future TKRs observed in the MR patients compared with the PM patients, which outweighs the short-term costs arising from an increased revision rate for MR when the lateral and medial menisci are combined. These findings suggest that, when appropriate, MR should be considered a cost-effective alternative to PM and may provide an opportunity for cost savings in both the short term and long term.

The findings in this study are broadly in line with those in the existing economic literature^14^, ^15^, ^16^, ^17^, ^18^ that suggests MR is the dominant or preferred strategy over a relatively short time horizon when compared with PM for other types of tears (e.g., root tears). In addition, much like other economic evaluations considering other tear types, this study found that cost-effectiveness is not sensitive to the age at treatment.

### Limitations

Regarding study limitations, the model could be further improved if there was more direct head-to-head clinical research exploring the differences in key outcomes between MR and PM for HCTs. In particular, the model could be improved with direct clinical evidence on the RR of OA progression and the subsequent occurrence of TKR. It should also be noted that all costs and utilities came from the general meniscal tear literature, and it might have been preferable to have had these inputs for HCTs. However, there are instances in which this might not be expected to have a material impact on the results of the model. For example, the RR of OA progression between MR and PM used data for general meniscal tears but other studies have found that the different contact pressures resulting from MR and PM may not differ between tear types.^28^^,^^29^ Our sensitivity analysis assumes a range of ±20% from the base value to account for possible differences in values, which may not reflect the true range of values for these parameters. The parameters included also reflect a payor’s perspective for this study and do not include societal implications. In addition, it should be noted that it was not possible to consider medial and lateral tears separately because of limitations in the HCT literature.

Another possible limitation of this study is that although it would have been possible to include multiple revision TKRs, it was decided not to implement this in the model. This decision was made because it might be difficult to attribute further revision TKRs to the primary treatment (i.e., PM or MR) rather than the subsequent TKR. Moreover, adding further revision TKRs would likely lead to a more favorable result for MR because this treatment resulted in fewer TKR procedures, and thus, it would be expected that this treatment pathway would require fewer revision TKRs. Finally, we note that this analysis was conducted from a US payor’s perspective and, as such, more research would need to be conducted to present results for other countries.

## Conclusions

Using a lifetime horizon, this study found that from a payor’s perspective, MR is a cost-saving intervention when compared with PM in patients with an HCT.

## Disclosure

The authors report the following potential conflicts of interest or sources of funding: N.A. is employed by Smith & Nephew. L.M.N. is employed by Smith & Nephew. R.J.S. is employed by Smith and Nephew and reports other funding from Smith & Nephew, during the conduct of the study and funding outside the submitted work. D.C.F. is a consultant for Smith & Nephew, DePuy Mitek, Vericel, and ConMed; receives research support from 10.13039/100009026Smith & Nephew, 10.13039/100008426DePuy Mitek, Vericel, ConMed, Moximed, Episurf, KCRN, 10.13039/100014927Anika Therapeutics, and Aesculap, outside the submitted work. All other authors declare that they have no known competing financial interests or personal relation

## Supplementary Data

ICMJE author disclosure forms

ICMJE Disclosure form for Searle
